# Black raspberry restores the expression of the tumor suppressor p120ctn in the oral cavity of mice treated with the carcinogen dibenzo[a,l]pyrene diol epoxide

**DOI:** 10.1371/journal.pone.0259998

**Published:** 2021-11-16

**Authors:** Douglas B. Stairs, Mary E. Landmesser, Cesar Aliaga, Kun-Ming Chen, Yuan-Wan Sun, Karam El-Bayoumy

**Affiliations:** 1 Department of Pathology, College of Medicine, The Pennsylvania State University, Hershey, Pennsylvania, United States of America; 2 Penn State Cancer Institute, College of Medicine, The Pennsylvania State University, Hershey, Pennsylvania, United States of America; 3 Department of Biochemistry and Molecular Biology, College of Medicine, The Pennsylvania State University, Hershey, Pennsylvania, United States of America; University of Central Florida College of Medicine, UNITED STATES

## Abstract

One of the major risk factors for head and neck squamous cell carcinoma (HNSCC) is tobacco smoke exposure, but the mechanisms that can account for disease development remain to be fully defined. Utilizing our HNSCC mouse model, we analyzed oral squamous cell carcinomas (OSCC) induced by the active metabolite of a common smoke constituent, dibenzo[a,l]pyrene diol-epoxide (DBPDE). Analyzing protein expression by either immunofluorescence or immunohistochemistry, we identified biologic processes that are dysregulated in premalignant and invasive cancer lesions induced by DBPDE. Interestingly, p120ctn expression is downregulated in both stages of the disease. In addition to decreased p120ctn expression, there was also increased proliferation (as measured by Ki67), inflammation (as measured by NFkB (p65) expression), neovascularization (as measured by CD31) and recruitment of Ly6G-positive immune cells as well as strong EGFR expression. We also examined the effect of the chemopreventive agent black raspberry (BRB) on p120ctn and EGFR protein expression in DBPDE treated mice. p120ctn, but not EGFR, protein expression increased in mice treated with BRB. Our results suggest that modulation of p120ctn may, in part, account for the mechanism by which BRB inhibits DBPDE induced OSCC in mice.

## Introduction

Head and neck squamous cell carcinoma (HNSCC), including oral cavity, oropharynx, nasopharynx, hypopharynx and larynx malignancies, accounted for 878,348 new cases and 444,347 deaths in 2020 [1]. Together, oral squamous cell carcinoma (OSCC) and oropharyngeal squamous cell carcinoma (OPSCC) are the most common HNSCC types resulting in an estimated 54,010 new cases and 10,850 deaths in the US in 2020 [2]. As recently reported in a thorough clinical review, it is clear that the prognosis and multimodal therapeutic options for patients with HNSCC can vary widely depending on epidemiologic factors, anatomical locations and stage of the disease [3]. Development of targeted therapy in HNSCC has been slow and even if treated successfully patients may be left with critical functional deficiencies including impaired abilities to eat, drink and vocalize effectively. The 5-year survival rate has remained unchanged at about 50% for decades and varies by race and stage of disease [2]. As classified by the International Agency for Research on Cancer (IARC), risk factors for carcinogenicity in humans for OSCC and, to a lesser extent, OPSCC include smoking, alcohol consumption, and poor oral hygiene [4]. Other factors such as betel quid, smokeless tobacco, and marijuana use, and certain dietary components have also been implicated [5]. Carcinogens from tobacco smoke exposure are known to induce DNA damage and mutagenesis. Over time, exposure to these carcinogens can result in histologically evident tissue damage that subsequently develops into dysplasia, carcinoma *in situ*, and ultimately, invasive SCC.

Despite understanding the development of the histologic changes that lead to HNSCC from smoke exposure, the molecular events that regulate these changes and aid in the development of an aggressive cancer are still not fully understood. Several oncogenes have been identified as drivers of OSCC such as PIK3CA and CyclinD1 as well as tumor suppressors such as p53, pRb and p120-catenin (p120ctn) [6, 7]. p120ctn is downregulated or lost in approximately 75% of human HNSCC and is associated with a poor prognosis [7]. We were the first to show that loss of p120ctn in the oral cavity of mice results in OSCC that is histologically identical to that of human OSCC [8]. Seventy percent of these mice develop well-differentiated squamous cancers by 12 months of age. Interestingly, the OSCC that develops occurs within a background of an active tumor microenvironment containing activated fibroblasts with significant desmoplasia and a chronic inflammatory response with increased myeloid derived suppressor cells. Because 30-40% of human HNSCC have PIK3CA activating mutations, we also explored how p120ctn could cooperate with mutant PIK3CA to induce HNSCC [9]. We demonstrated that p120ctn downregulation combined with PIK3CA mutations can transform oral keratinocytes and regulate invasion through MMP1 induction. Thus, p120ctn loss is not only important as a marker of a poor prognosis in oral cancer, it is also a major driver of the carcinogenic process. Relevant to HNSCC therapy, loss of p120ctn also induces resistance to Cetuximab, a targeted EGFR therapy frequently used to treat local-regional HNSCC [10].

As mentioned above, smoking is a major risk factor for the development of HNSCC. Our research team has previously demonstrated that the smoke carcinogen dibenzo[def,p]chrysene, also known as dibenzo[a,l]pyrene (DBP), and its active metabolite DBP diol-epoxide (DBPDE), induced DNA damage in the mouse oral cavity with subsequent gene mutations, including p53 mutations, that lead to tumorigenesis [11, 12]. DBP and DBPDE also induces epigenetic changes including hypomethylation of the Fgf3 promoter [13]. Inflammation is also induced in these mice and is exemplified by increases in Cox2 gene expression as well as several inflammatory cytokines such as Ccl22 and Cxcl10 [14]. Our team also demonstrated that feeding black raspberry (BRB) to both DBP and DBPDE treated mice inhibits tumor formation and altered several molecular targets in a manner consistent with its preventive/therapeutic action for HNSCC [15, 16].

Interestingly, it has been shown that adherens junctions can be lost following smoke treatment of lung epithelium *in vitro* [17]. Therefore, in the present report, we aimed to investigate the effects of DBPDE on p120ctn expression and the further impact of BRB *in vitro* and *in vivo*. We also explored whether there are similarities between tumor development as a result of chemically induced (DBPDE) OSCC and that induced via genetic alterations (p120 deletion).

## Materials and methods

### Animal use, chemical carcinogen and black raspberry treatments

The Institutional Animal Care and Use Committee (IACUC) at the Penn State College of Medicine approved all animal studies. Mice were housed under a 12-hr light/dark cycle and fed ad libitum. L2-Cre;p120ctn loxP/loxP mice were generated and genotyped as previously described [8]. Archived oral tumors from our previous studies [15] were employed in the present investigation. The details of the mouse bioassay testing the effect of topical applications of DBPDE into the oral cavity on tumorigenesis and the protective effect of BRB have been published [15]. Briefly, two groups of B63F1 mice (30/group) at the age of 8 weeks received 3 nmol of DBPDE in DMSO, 3 times a week for 38 weeks; the dose was selected based on our previous studies [12]. One group of mice was fed AIN-93M control diet and the other group was fed AIN-93M diet containing 5% BRB starting 2 weeks prior to the initiation of carcinogen treatment. At termination, mice were sacrificed by CO_2_ asphyxiation with either cervical dislocation or cardiac exsanguination and organ removal as secondary methods of euthanasia, Oral tumors were collected, processed, and examined histopathologically for tumor classification and for assessing protein expression by IHC/IF.

### Black raspberry preparation

Black Raspberry preparations were generated as described previously [18, 19]. Briefly, freeze-dried BRB powder was purchased from Decker Farms, Inc and BerriProducts, LLC. For treatment in animals, BRB powder was added to the AIN-93M diet to obtain a 5% w/w mixture of BRB. BRB was incorporated into the diet using a Hobart mixer for 20 minutes. For BRB extract (BRBE), BRB powder was dissolved in 80% ethanol-H_2_O and used at a concentration of 160ug/ml in culture media.

### Cell culture

Immortalized normal oral keratinocytes (NOK-hTERT cells) were cultured as previously described in keratinocyte serum free media (Invitrogen) [9]. For BRBE and DBPDE treatments, cells were passage the day before treatment for a target confluence at treatment of approximately 50-60%. For BRBE, cells were treated with 160ug/ml of BRBE. For DBPDE treatment, cells were treated with freshly dissolved DBPDE at a final concentration of 70nM. Doses of DBPDE and BRBE were selected based on our previous studies [18, 20].

### Western blot analysis

Cell protein lysates were harvested by rinsing plates in PBS and using a lysis buffer consisting of Tris-HCL, NaCL, NP-40 and phosphatase and protease inhibitors as described previously [9]. Western blotting was performed as described previously [21]. Briefly, a 10% denaturing acrylamide gel was used to separate 10ug of the protein lysates and then transferred to a PVDF membrane. Primary antibodies, p120ctn (BD Transduction Labs #610134 – 1:10,000 dilution) and b-actin (Sigma-Aldrich #A5316 – 1:10,000 dilution), were incubated overnight at 4°C. Secondary antibody, Goat anti-mouse IgG (H+L) (Millipore #AP124P – 1:5000 dilution), was incubated at room temperature for 1 hour. ECL Prime (Cytiva Amersham #RPN2232) was used to develop the western blot and was read on a FluorChem R System (ProteinSimple).

### Immunohistochemistry/Immunofluorescence (IHC/IF)

IHC was performed on paraffin sections using the Roche Benchmark Ultra Autostainer. Antigen retrieval method and antibody incubation times were optimized for each antibody (see below). Secondary antibodies were incubated for 20 minutes. After staining, slides were removed from the autostainer and coverslipped with Permount (Fisher Scientific). Primary antibodies: NFkB (#4764, Cell Signaling Technology) incubation conditions: CCL2 retrieval solution for 32 min, 1:100 primary antibody for 1 hour, CD31 (#77699, Cell Signaling Technology) incubation conditions: CCL1 retrieval solution for 24 min, 1:100 primary antibody for 20 minutes, Ly6G (#87048, Cell Signaling Technology) incubation conditions: CCL1 retrieval solution for 32 min, 1:200 primary antibody for 20 minutes, EGFR (#71655, Cell Signaling Technology) incubation conditions: CCL1 retrieval solution for 32 min, 1:500 primary antibody for 32 minutes, Ki67 (#RM-9106-S0; Thermo Scientific) incubation conditions: CCL1 retrieval solution for 48 min, 1:100 primary antibody for 32 minutes.

IF was performed as described previously [21]. Briefly, antigen unmasking was performed with the Retriever (Electron Microscopy Sciences, Hatfield, PA) in 10 mmol/L sodium citrate buffer, pH 6.0. Endogenous peroxidase activity was blocked by incubation with 3% hydrogen peroxide for 6 minutes. Slides were incubated in the p120ctn primary antibody (610134; BD Transduction) overnight at 4°C. Secondary antibody Alexa Fluor 488-anti-mouse (A-11001; Invitrogen) incubation was for 1 hour at room temperature. Slides were coverslipped using VectaShield HardSet Antifade mounting medium (H1400; Vector Laboratories).

### Quantification of immunofluorescence images

Sections were imaged and photographed with an Olympus BX53 microscope (Olympus America). All images were acquired at 2 second exposure time with all settings identical. All samples had an identical preset curve applied to them to remove background and increase the dynamic range of the signal intensity present. The preset curve was generated using a normal histology image, maintained intensities in a linear range, and was applied to all samples regardless of experimental condition. All IF samples were analyzed with PhotoShop using the measurement log to obtain the mean and maximum gray value intensities for each image. Microsoft Excel was used for data analysis, graph generation and statistical analysis (one-tailed Student’s t-test assuming normal distribution with equal variance).

## Results

### p120ctn expression decreases in oral tumors of mice treated with DBPDE

**Fig 1:**
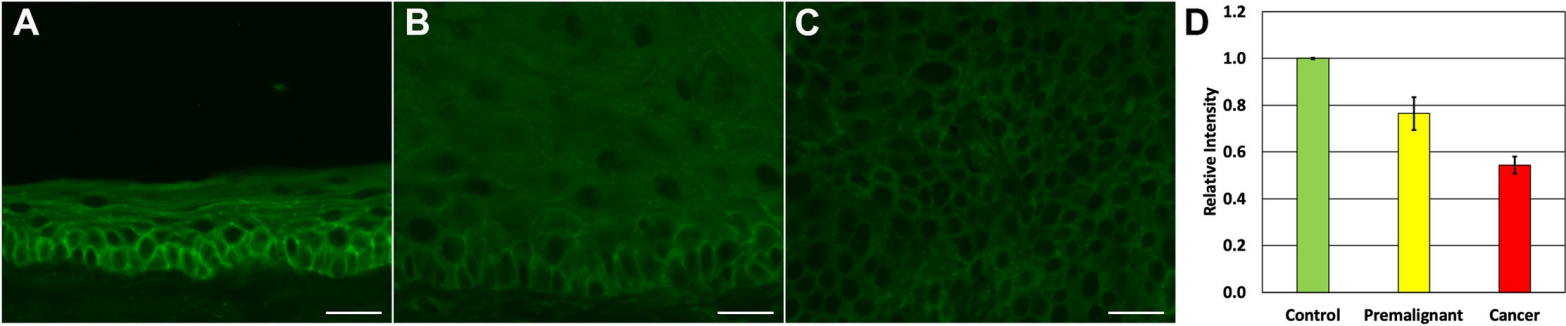
Decreased membranous expression of p120ctn in DBPDE-induced premalignant and cancer lesions. Staining for p120ctn was performed on oral mucosal tissue harvested from C57Bl/6 mice treated with DBPDE (n = 15) or DMSO (n = 10) as control mice. Representative samples are shown for: (A) Normal oral tissue from control mice, (B) premalignant tissue with decreased p120ctn expression, (C) invasive tumor with decreased p120ctn expression. (D) Quantification of p120ctn mean fluorescent intensity. Scale Bar = 20uM.

Given that p120ctn is lost, down-regulated or mislocalized in approximately 75% of HNSCC [7], we set out to examine whether p120ctn protein expression is also lost in pre-malignant and OSCC lesions induced by DBPDE, the ultimate carcinogenic metabolite of the tobacco smoke constituent DBP. Oral cavity tissue was previously harvested from 15 DBPDE treated mice and analyzed by a veterinary pathologist for tumor formation [12]. H&E analysis of these fifteen samples demonstrated that nine mice had OSCC, five mice had premalignant lesions and one had no visible lesions in the tissue sections analyzed. p120ctn protein staining was performed on these tissues by immunofluorescence (IF). Staining intensity of p120ctn in OSCC and pre-malignant lesions was assessed in mice in relation to adjacent normal epithelium as well as to DMSO treated mice and scored as increased, decreased, or no change in staining intensity (Table 1). The single DBPDE treated mouse with normal histology had good membrane localization of p120ctn in the basal layers of its epithelium as previously reported for normal p120ctn expression in the oral cavity of mice (Fig 1A) [8]. No mice had increased staining intensity in premalignant or cancer lesions. In premalignant lesions, three of five mice had decreased, complete loss or mislocalization of p120ctn expression (Fig 1B). Of the nine invasive cancers, seven had either decreased or complete loss of p120ctn protein expression (Fig 1C). Mean fluorescent intensity was quantified (n = 3 for each group) in control tissues with normal histology, premalignant tissues with p120ctn loss and in cancers with p120ctn loss (Fig 1D) demonstrating significant down-regulation of p120ctn in premalignant lesions and invasive cancers (p<0.05).

**Table 1:** p120ctn protein expression changes in DBPDE mice (n = 15) versus controls (DMSO) (n = 10).

Pathologic diagnosis	Number of mice	No change	Decreased
**Control mice (DMSO)**	10	10	0
**Normal**	1	1	0
**Premalignant**	5	2	3
**Invasive Cancer**	9	2	7

### Increases in proliferation and inflammation in DBPDE treated mice

**Fig 2:**
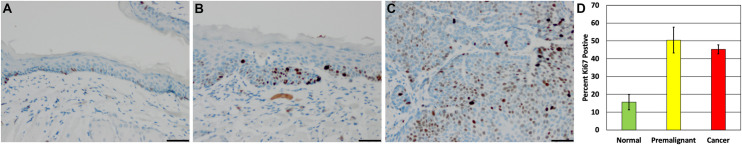
Ki67 increases in DBPDE treated mice. (A) Ki67 staining of normal tissues demonstrates a low proliferative index in the basal layers of the epithelium. (B) Dysplastic lesions have an increase in proliferation that is expanded beyond just the basal layer. (C) Regions of invasive cancer have wide-spread increases in proliferation. Scale bar = 50uM. n = 15. (D) Quantification of the percentage of Ki67 positive cells measured by counting 40x images (n = 3).

Proliferation was assessed by Ki67 staining. Positive Ki67 staining was seen in isolated cells spaced somewhat evenly throughout the basal layer of normal oral mucosa tissue (Fig 2A). In contrast, pre-malignant and invasive squamous cancer lesions had increases in Ki67 staining. In premalignant lesions, an increased percentage of Ki67 positive cells was observed in basal and suprabasal layers (Fig 2B). As cells fully differentiate toward the lumen of these premalignant lesions, no Ki67 positive cells were detected. In invasive squamous cancer lesions, Ki67 positive cells were abundant and found throughout the tumor (Fig 2C). The percentage of Ki67 cells present in the epithelia or cancer cells were quantified (n = 3 for each group) in control tissues with normal histology and in premalignant and invasive cancers tissues following DBPDE treatment demonstrating a significant increase in proliferation in premalignant lesions and invasive cancers (p<0.05) (Fig 2D).

**Fig 3:**
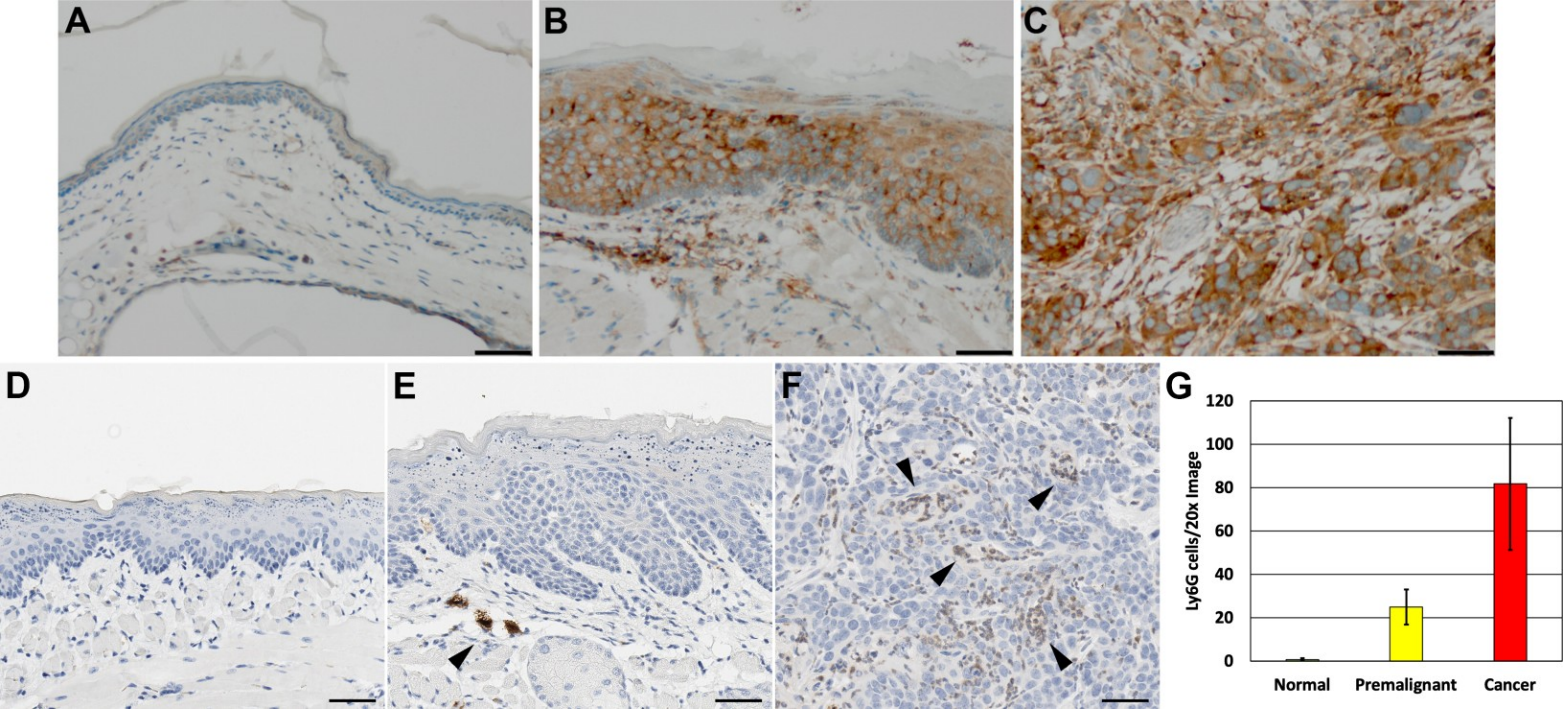
Increased NFkB and immune cell presence in DBPDE-induced mice. (A) Normal oral epithelium in DBPDE treated mice are negative or weakly positive for total p65 NFkB protein levels as measured by IHC staining. (B) A representative image of a dysplastic epithelium from DBPDE-treated mice that has increased NFkB staining. (C) NFkB staining is increased in invasive cancers of DBPDE-treated mice. Ly6G was used as a marker for MDSCs, primarily of the neutrophil precursor lineage. (D) Normal oral epithelia do not have a significant presence of Ly6G positive cells. (E) DBPDE-induced dysplasia and (F) invasive cancers do have an increased number of Ly6G-positive cells suggesting a role for MDSCs in tumor development in DBPDE treated mice. Scale bar = 50uM. n = 15. (G) Quantification of Ly6G recruitment measured by counting Ly6G cells/20x image (n = 3).

Many immunogenic cancers have activated NFkB expression, therefore, we assessed whether DBPDE induced tumors had increased NFkB (p65) expression. Normal epithelia had little to no detectible total NFkB (p65) protein staining (Fig 3A). In contrast, strong IHC staining for NFkB was detected in premalignant lesions (Fig 3B) as well as in invasive cancers (Fig 3C). As with many epithelial cancer types, HNSCC is influenced by the type of immune cells present in its microenvironment [22]. Specifically, myeloid lineage cells including myeloid-derived suppressor cells (MDSCs), and pro-tumorigenic neutrophils and macrophages. Suppression of these predominantly neutrophil lineage cells lead to decreased tumor formation. To determine when during cancer development these cells are recruited to the tissue microenvironment, Ly6G IHC was performed in our DBPDE treated mice. The stroma surrounding premalignant lesions and invasive cancers were assessed for the presence of Ly6G positive cells. Under normal conditions, Ly6G positive cells are not seen in or around oral epithelium (Fig 3D). In DBPDE-induced premalignant lesions, there is a recruitment of several strongly positive Ly6G cells to the microenvironment (Fig 3E). In invasive cancers, Ly6G positive cells can be seen in the stromal regions within the tumor mass itself (Fig 3F). The number of Ly6G cells present was quantified (n = 3 for each group) in control tissues with normal histology and in premalignant tissues and invasive cancers following DBPDE treatment demonstrating significant recruitment of Ly6G positive cells to premalignant lesions and invasive cancers (p<0.05) (Fig 3G).

### Increased vasculature in DBPDE treated and p120ctn null mice

**Fig 4:**
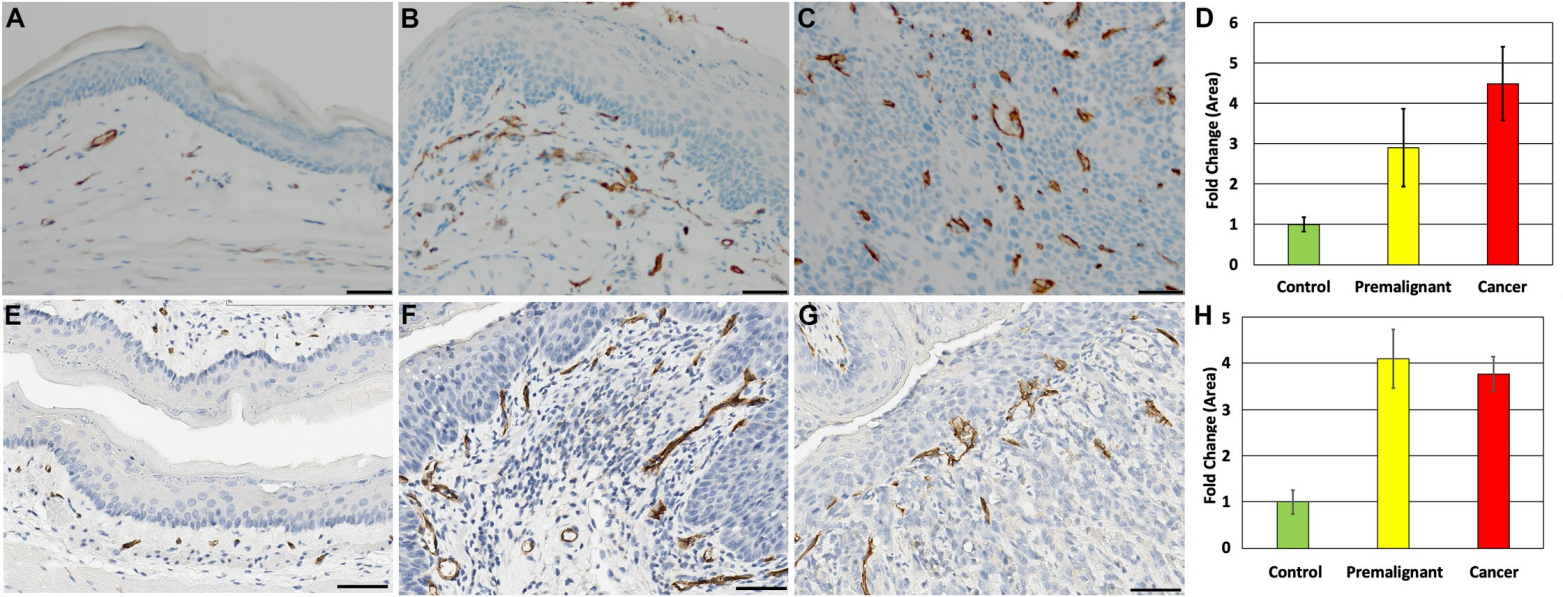
Increased vascular density surrounding DBPDE-induced lesions as well as in p120ctn-null lesions. CD31 staining reveals that there is an increase of vascular development (neovascularization) in the oral tissues of DBPDE-treated mice. (A) CD31 staining of vasculature reveals a regular, single layer of vasculature below the oral cavity epithelium. (B) CD31 increases occur very early and can be seen adjacent to in hyperplastic/mildly dysplastic epithelium. (C) In invasive cancer regions, the vasculature can be seen throughout the tumor. n = 15 for DBPDE mice and n = 10 for controls. (D) Quantification of CD31 staining in DBPDE treated mice as measured by fold difference in CD31 stained area (n = 3). (E) Normal epithelia have a regular vascular pattern underlying the basal layer as measured by CD31. (F) As dysplasia progresses, the blood vessels become very prominent and extend to the tips of the stromal stalks that form. (G) In invasive cancer lesions, the vasculature is fully integrated with the tumor cells. Scale bar = 50uM. n = 5 for p120ctn knockout mice and controls. (H) Quantification of CD31 staining in p120ctn knockout mice as measured by fold difference in CD31 stained area (n = 3).

Given the increases seen in immune cell recruitment to DBPDE induced lesions, we examined whether other tumor microenvironment components were induced during tumor formation. CD31 staining was employed to visualize vasculature. In normal oral tissue, a thin layer of evenly spaced CD31 positive blood vessels below the epithelium can be visualized (Fig 4A). These blood vessels are not immediately adjacent to the epithelium but rather are found approximately 10-20um below the basement membrane. In DBPDE-induced premalignant lesions, this layer is significantly expanded and CD31 positive blood vessels can be detected immediately adjacent to the epithelium (Fig 4B). In invasive cancer lesions, the CD31 positive vasculature is integrated throughout the tumor (Fig 4C). The abundance of vasculature present in DBPDE treated mice was quantified (n = 3 for each group) in control tissues with normal histology and in premalignant tissues and invasive cancers following DBPDE treatment demonstrating significant increases in vascularization surrounding premalignant lesions and invasive cancers (p<0.05) (Fig 4D).

The induction of an active tumor microenvironment in premalignant and invasive cancer lesions of DBPDE treated mice resemble that seen in a genetically engineered mouse model of oral cancer, the p120ctn knockout mouse with loss of p120ctn targeted to the oral cavity and esophagus [8]. These mice recruit MDSCs that are predominantly of a neutrophil precursor lineage by morphology analysis and inhibition of inflammation prevents tumor formation. Given that p120ctn is decreased in DBPDE treated mice (Fig 1 and Table 1), we investigated the vascular patterns in p120ctn knockout mouse tissue. As described above, a regular vascular pattern is seen below normal epithelium of control mice (Fig 4E). In premalignant lesions of p120ctn knockout mice, we see an increase in the number of CD31 positive cells and in addition to their presence immediately adjacent to the epithelium (Fig 4F), just as seen in the present study with DBPDE treated mice. In invasive cancers induced by p120ctn loss, CD31 positive cells are integrated within the tumor cells throughout the lesions (Fig 4G). The abundance of vasculature present in p120ctn knockout mice was quantified (n = 3 for each group) in control tissues with normal histology and in premalignant tissues and invasive cancers following DBPDE treatment again demonstrating significant increases in vascularization surrounding premalignant lesions and invasive cancers (p<0.05) (Fig 4H).

### Black raspberry (BRB) treatment increases p120ctn expression

**Fig 5:**
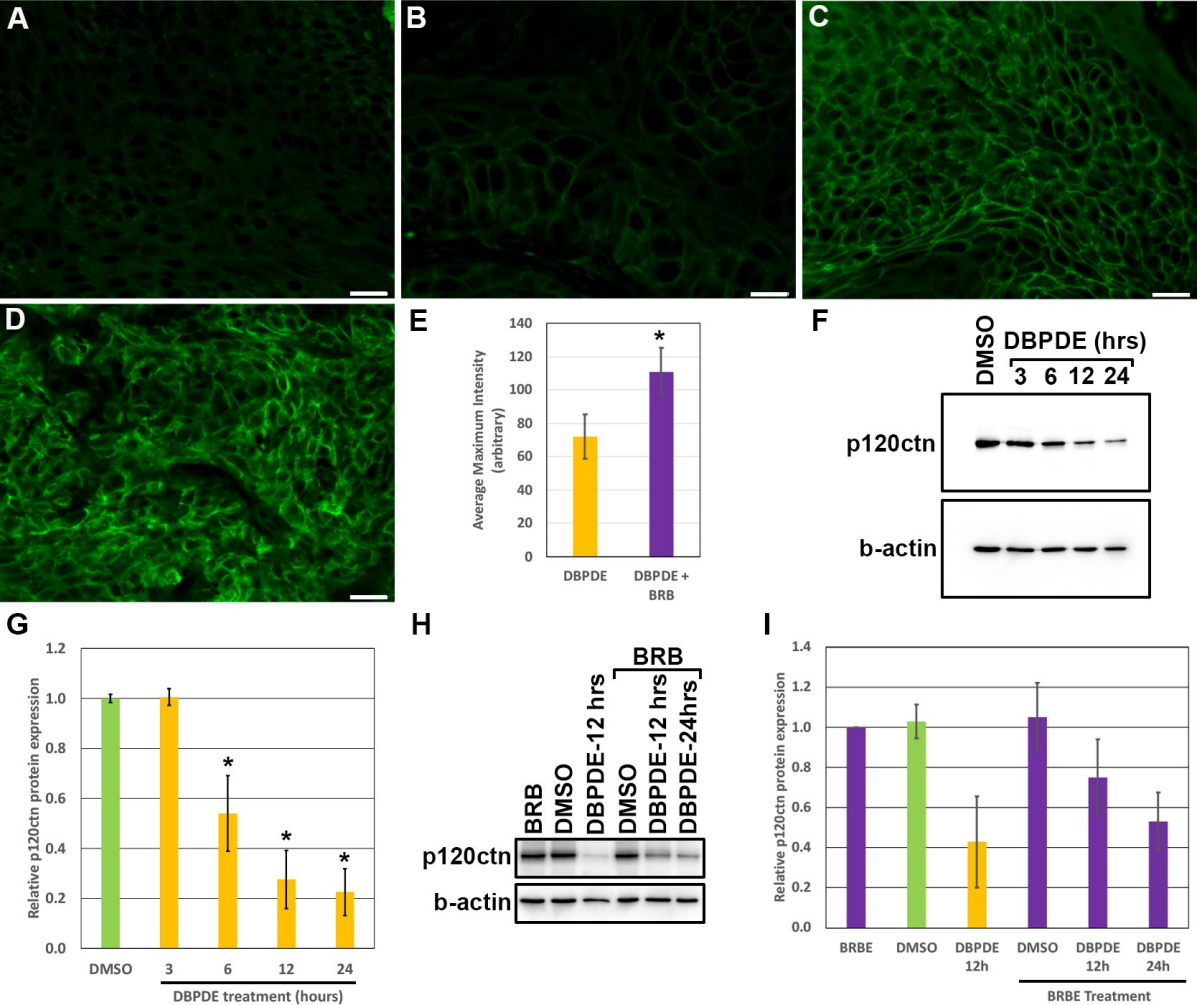
p120ctn expression increases in BRB treated mice. IF for p120ctn was performed on oral mucosal tissue samples harvested from C57Bl/6 mice treated with DBPDE with BRB (n = 10) and without BRB (n = 7) administration. Mice treated with DBPDE alone, panels (A) and (B), have a relatively low expression level of p120ctn while DBPDE + BRB treated mice have relatively increased levels of p120ctn, panels (C) and (D). Panels (A) and (C) represent papillomas while panels (B) and (D) represent invasive cancer. Scale bar = 20uM. (E) Quantification of the mean intensity of p120ctn IF from representative images of papillomas and invasive cancers from all DBPDE treated mice, with and without BRB. (F) Western blot analysis for p120ctn protein levels in DBPDE treated NOK-hTERT cells. b-actin serves as a loading control (G) Quantification of the p120ctn band intensities from western blot analysis, normalized to b-actin (n = 3). (H) Western blot analysis for p120ctn protein levels in DBPDE and/or BRBE treated NOK-hTERT cells. b-actin serves as a loading control (I) Quantification of the p120ctn band intensities from western blot analysis, normalized to b-actin (n = 3).

Previous work has shown that black raspberry (BRB) treatments can function as a nutraceutical therapy for HNSCC [20, 23]. In mice treated with DBPDE we found that diet containing 5% BRB resulted in a significant reduction of tumor formation [15]. Given that p120ctn can alter EGFR therapy in HNSCC [10] and, as shown in Fig 1, p120ctn is downregulated following DBPDE treatment, we set out to determine if BRB treatment altered p120ctn expression in DBPDE-induced tumors. Oral cavity tissue was previously harvested from DBPDE treated mice with and without BRB administration. p120ctn expression was assessed in papillomas and invasive tumors in the oral cavity of DBPDE treated mice fed with BRB (n = 10) or without BRB (control group, n = 7). Similar to Fig 1 and Table 1, p120ctn expression was low in DBPDE induced papillomas and invasive cancer (Fig 5A and 5B, respectively). Interestingly, DBPDE+BRB treated mice with papillomas and invasive cancer have increased p120ctn expression in papillomas and invasive cancer (Fig 5C and 5D, respectively) relative to DBPDE-only treated mice. Quantification of maximum p120ctn staining intensity, and therefore membranous staining, of p120ctn staining intensity demonstrates a statistically significant increase in p120ctn expression in mice treated with DBPDE and fed BRB (Fig 5E).

To better establish a direct relationship between DBPDE, BRB, and p120ctn, we treated NOK-hTERT cells with DMSO, as a vehicle control, or with DBPDE. Similar to the effects seen *in vivo*, DBPDE treatment reduced p120ctn protein levels compared to vehicle control starting as early as 6 hours after treatment (Fig 5F). Quantification of western blot band intensities demonstrates a statistically significant difference (p-value < 0.05) in p120ctn protein expression between DMSO versus 6, 12 and 24 hours after DBPDE treatment (Fig 5G). Interestingly, BRB extract (BRBE) alone did not have a significant effect on p120ctn expression in NOK cells versus DMSO treatment (Fig 5H). However, as seen with our *in vivo* results, BRBE treatment in cell culture attenuated decreases in p120ctn protein expression in cells also treated with DBPDE (Fig 5H). Quantification of p120ctn western blot band intensities demonstrates a significant difference (p-value < 0.05) between vehicle (DMSO) and 12 hours post DBPDE treatment as well as between 12 hours post DBPDE treatment and 12 hours post DBPDE + BRB treatment (Fig 5I). These data suggest a direct cause and effect relationship between both BRB and DBPDE and p120ctn protein expression. Furthermore, it is possible that the anti-tumor effects of BRB may, at least partly, be mediated by its effects on the tumor suppressor p120ctn.

### High EGFR expression in DBPDE treated mice

**Fig 6:**
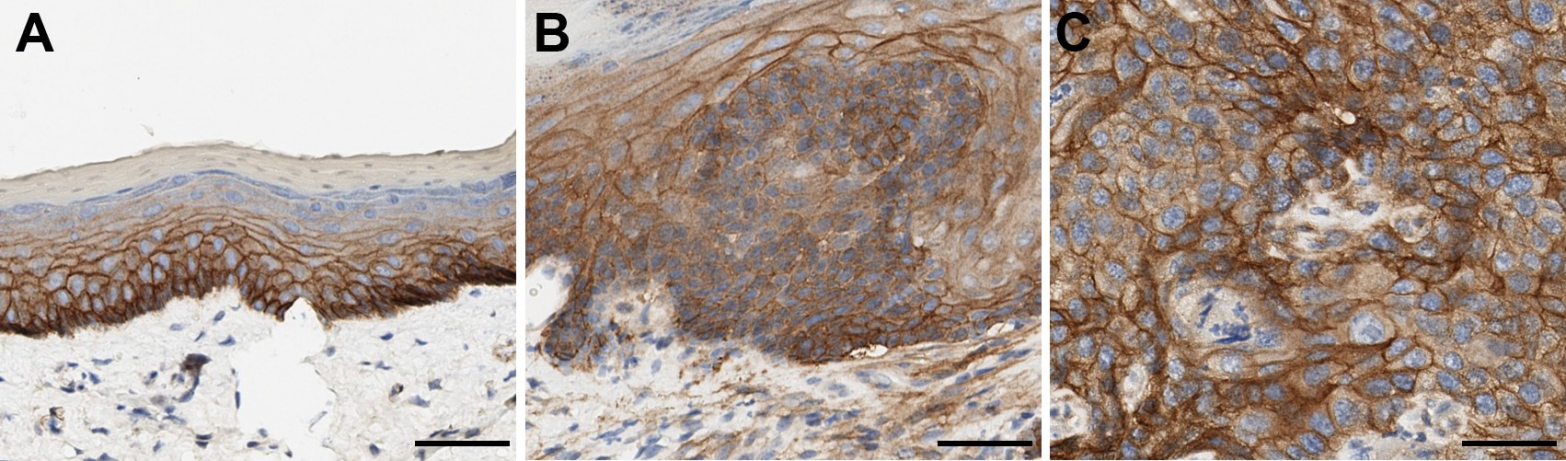
High EGFR expression in DBPDE-treated mice. (A) EGFR expression in normal epithelia is highest in the basal layer and decreases as cells become more differentiated and superficial. (B) EGFR expression is high in hyperplastic and dysplastic epithelium. (C) EGFR expression is high in invasive cancers. n = 15. *p<0.05.

Because we have previously shown that decreased p120ctn expression causes EGFR therapy resistance [10], we wanted to determine EGFR expression levels in DBPDE treated tumors and whether BRB treatment altered its expression. In normal epithelium, EGFR is strongly expressed in the basal layers, and expression decreases as the epithelium differentiates and migrates toward the superficial layers (Fig 6A). Analysis of premalignant and invasive cancers of DBPDE treated mice revealed that EGFR expression remains high in dysplastic and invasive cancer cells (Fig 6B and 6C, respectively). Analysis of EGFR expression in DBPDE treated mice fed a diet containing BRB was performed in a manner similar to p120ctn IF. IHC of EGFR in papillomas (S1A and S1C Fig) and invasive cancers (S1B and S1D Fig) in DBPDE treated mice was performed. Strong staining was seen in BRB fed mice (S1C and S1D Fig) but the intensity was comparable to those fed control diet without BRB (S1A and S1B Fig). Therefore, BRB treatment did not alter the strong EGFR expression seen in invasive oral cancers induced by DBPDE exposure.

## Discussion

Avoidance of risk factors has been only partially successful in preventing HNSCC, largely because of the addictive power of highly relevant etiological agents such as tobacco [5]. Therefore, our ultimate goal is to develop novel or improved approaches to prevention as well as to discover markers for early detection to manage and control this disease. Preclinical animal models that 1) employ carcinogens found in tobacco smoke, 2) reflect tumor heterogeneity, and 3) accurately reflect the cellular and molecular changes in the multi-step process of oral carcinogenesis in humans could provide a realistic platform to achieve our goal. Thus, we introduced an OSCC mouse model using the tobacco smoke constituent DBP and its ultimate carcinogenic metabolite diol-epoxide, DBPDE [11, 12]. While the carcinogenicity of these agents in the oral cavity of mice has been reported, the mechanisms and pathways leading to tumor formation remain to be fully defined [5]. Therefore, in the present study we focused on exploring the effects of DBPDE on molecular targets that have been shown to be critically involved in the development of human HNSCC. Histologically confirmed archived oral cavity tissues of mice treated with DBPDE [15] were used in the current study. We initially focused on the effects of DBPDE on p120ctn protein expression since it has been shown to be downregulated or mislocalized in approximately 75% of HNSCC [7]. Our preclinical data demonstrate that DBPDE significantly reduced expression of p120ctn in the oral tumors of mice and our results are consistent with those observed in human HNSCC [7].

In DBPDE-induced oral cancer in mice we found strong NFkB (p65) protein staining in both premalignant and invasive cancers but not in normal epithelia. Activation of NFkB is known to activate multiple genes involved in inflammation and cell proliferation [14]. In fact, we reported previously that both DBP and DBPDE upregulate COX-2 protein expression as well as several inflammatory genes [11, 12, 14]. Apart from the effects of DBPDE on inflammatory markers, it is clear that this carcinogen is capable of increasing cell proliferation, as indicated by increased Ki67 staining, in both premalignant and invasive lesions.

The type of immune cells in the tumor microenvironment can influence many epithelial cancers including HNSCC [22]; examples include MDSCs and pro-tumorigenic neutrophil and macrophages. In the present study, the results demonstrate that in contrast to normal epithelia, Ly6G positive cells can be seen in invasive cancers. Of note, literature data showed that OSCC induced by the synthetic carcinogen 4-Nitroquinoline N-oxide (4NQO), not found in tobacco smoke, also increased Ly6G positive cells present in the tumor microenvironment [24]. Taken together, our results using DBPDE and those reported in the literature using 4NQO [24] are consistent with a model whereby recruitment of pro-tumorigenic myeloid cells, predominantly of neutrophil lineage, occurs before invasive cancer develops which may aid in the carcinogenesis process.

We further examined other components in the tumor microenvironment in our DBPDE induced oral lesions in mice focusing on CD31 staining to visualize changes in the vasculature. Our results indicate that there are distinct differences in CD31-positive vasculature between normal, premalignant and invasive cancer tissues. We next compared the changes in CD31 identified in this study following chemically (DBPDE) induced oral cancer with that observed in the p120ctn knockout mouse [8]. The results demonstrate that timing of neovascularization and vascular patterning is similar in the two mouse models, suggesting the possibility that tumorigenesis can occur by similar mechanisms.

Plants and herbs have historically been used to treat medical problems and a percentage of modern medicines have been extracted from plants [23]. Our laboratory used BRB as a whole food approach for cancer prevention since it is safe and more appealing to administer to humans [20]. We showed that BRB reduces oxidative stress in human oral leukoplakia cells [25]. Furthermore, we demonstrated that BRB inhibited OSCC induced by DBP or DBPDE via genetic and epigenetic alterations [15, 16]. We also found that BRB was associated with a significant hypomethylation of the fibroblast growth factors (eg. *Fgf3* gene) which are involved in epithelial-mesenchymal transition (EMT) pathways [16]. Our results are of clinical significance as frequent amplification of the *Fgf3* gene has been found in human tumors including HNSCC [13].

Previously published clinical trials that utilize BRB provided strong support for the benefits of BRB in patients with dysplastic lesions [26, 27]. These studies demonstrate histologic regression in about 60% of lesions and a significant reduction in loss of heterozygosity at tumor suppressor gene *loci*. In a phase 0 clinical trial, it was reported that BRB administered 14 days before surgery suppressed proinflammatory and prosurvival cancer markers in oral cancer patients [28]. Specifically, following BRB administration the expression of important oncoproteins such as EGFR and NFkB were significantly reduced. We have shown previously that decreased expression of p120ctn can cooperate with EGFR to promote carcinogenesis and can cause EFGR therapy failure [10]. In the present studies we showed that BRB significantly increased p120ctn expression in mice treated with DBPDE. Whether BRB can restore p120ctn expression via epigenetic regulation, as observed with *Fgf3* gene [16], remains to be explored. The enhancing effect of BRB on p120ctn expression led us to examine its effect on EGFR expression. Our results indicated that BRB did not alter the levels of EGFR expression in oral tissues of mice treated with DBPDE. These data demonstrate that p120ctn is sensitive to BRB treatment while EGFR is not. Furthermore, our data strongly suggest that cancer patients receiving EGFR therapy should be screened for p120ctn protein expression in their tumors to assess the potential efficacy of the targeted therapy. Collectively, our results indicate the need to explore the effect of BRB in combination with anti-EGFR therapy (Cetuximab) initially in our animal models and ultimately in the clinic.

## Conclusions

**Fig 7:**
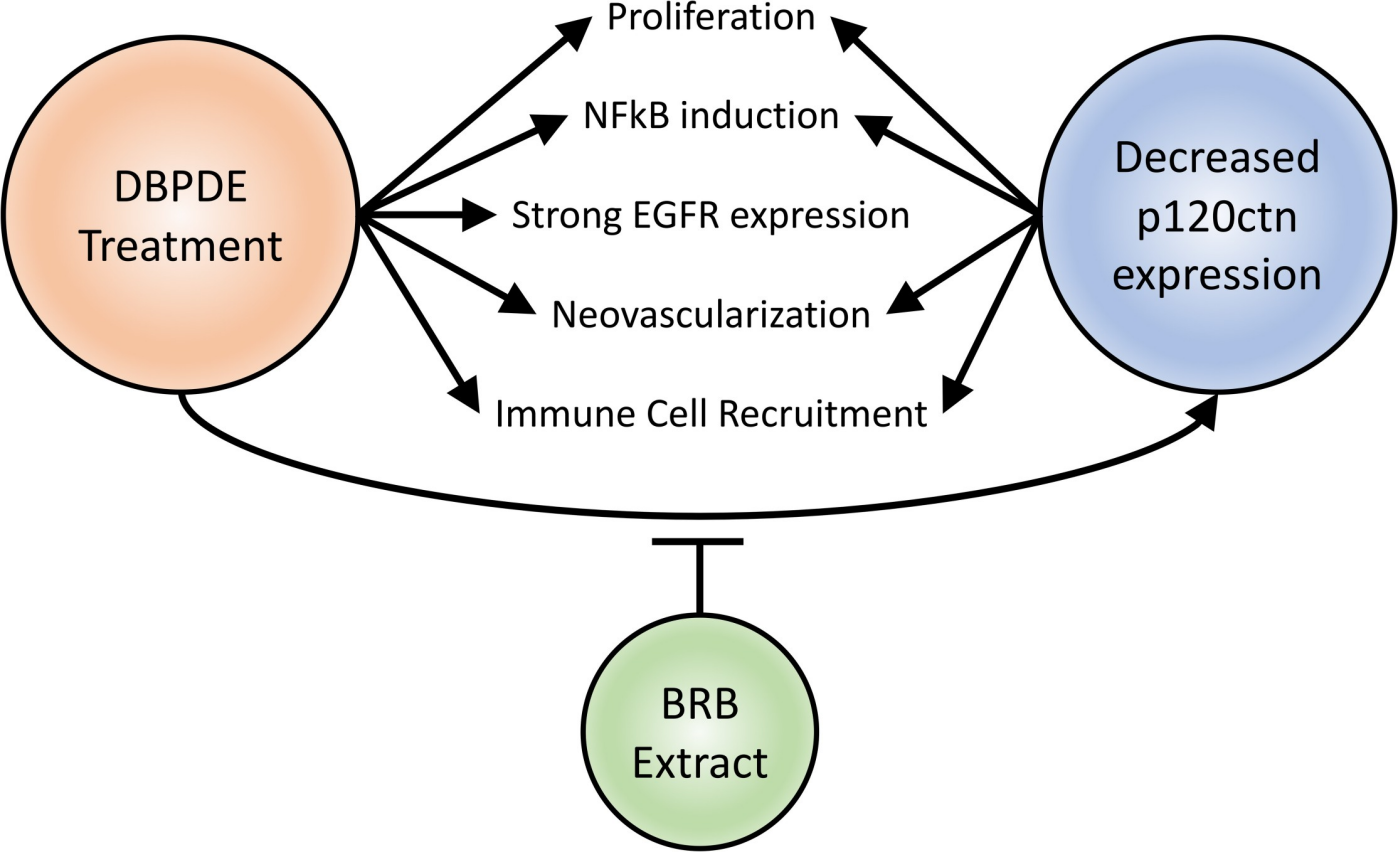
Schematic of the effects DBPDE treatment has on the tumor and its microenvironment. DBPDE treatment induced signaling changes in cells of premalignant and invasive cancer lesions resulting in increased NFkB and EGFR expression, increased proliferation, and reduced p120ctn expression. In the microenvironment, increased Ly6G cell (neutrophil lineage) recruitment and neovascularization was detected. These changes, except for EGFR expression, are common between DBPDE treatment and p120ctn decreased expression.

In summation, we have demonstrated that DBPDE induces many changes in the epithelium of the oral cavity as well as in its tissue microenvironment (Fig 7). DBPDE was shown, for the first time, to decrease p120ctn expression and increase proliferation as well as NFkB (p65) and EGFR expression. These changes occur in premalignant lesions and suggest they are early events that may drive carcinogenesis in this setting. Likewise, in the microenvironment, DBPDE treatment also induced neovascularization and Ly6G positive immune cell recruitment. Interestingly, we also have seen many of these changes with genetic deletion of p120ctn in the mouse oral cavity. This suggests, with the exception of EGFR expression, that p120ctn could be a major regulator of these pro-tumorigenic responses following DBPDE treatment. This also highlights the significance of BRB treatment attenuating the loss of p120ctn in DBPDE-induced tumors. Because p120ctn is a regulator of EGFR therapy efficacy, it will be important to investigate combined EGFR therapy with BRB treatment as a mechanism to negate the resistance induced by decreased p120ctn expression.

## Supporting information

S1 Fig EGFR expression does not change in BRB treated mice. IHC staining for EGFR was performed on oral mucosal tissue samples harvested from C57Bl/6 mice treated with DBPDE with BRB (n = 10) and without BRB (n = 7) administration. Panels (A) and (C) represent papillomas while panels (B) and (D) represent invasive cancer. Mice treated with DBPDE alone, panels (A) and (B), or with BRB, panels (C) and (D), have a high EGFR expression. Of note, is the heterogeneous appearance of expression in both papillomas and invasive cancers, regardless of BRB treatment or not. Scale bar = 50uM.
(TIF)

## References

[1] Sung H, Ferlay J, Siegel RL, Laversanne M, Soerjomataram I, Jemal A, et al. Global cancer statistics 2020: GLOBOCAN estimates of incidence and mortality worldwide for 36 cancers in 185 countries. CA Cancer J Clin. 2021. Epub 2021/02/05. doi: 10.3322/caac.21660 33538338

[2] Siegel RL, Miller KD, Jemal A. Cancer statistics, 2020. CA Cancer J Clin. 2020;70(1):7–30. Epub 2020/01/09. doi: 10.3322/caac.21590 . 31912902

[3] Chow LQM. Head and Neck Cancer. N Engl J Med. 2020;382(1):60–72. Epub 2020/01/02. doi: 10.1056/NEJMra1715715 . 31893516

[4] IARC. Monographs on the Identifiation of Carcinogenic Hazards to Humans: https://monographs.iarc.fr/wp-content/uploads/2019/07/Classifications_by_cancer_site.pdf. July, 2019.

[5] El-Bayoumy K, Christensen ND, Hu J, Viscidi R, Stairs DB, Walter V, et al. An Integrated Approach for Preventing Oral Cavity and Oropharyngeal Cancers: Two Etiologies with Distinct and Shared Mechanisms of Carcinogenesis. Cancer Prev Res (Phila). 2020;13(8):649–60. Epub 2020/05/22. doi: 10.1158/1940-6207.CAPR-20-0096 ; PubMed Central PMCID: PMC7415541. 32434808 PMC7415541

[6] Cancer Genome Atlas N. Comprehensive genomic characterization of head and neck squamous cell carcinomas. Nature. 2015;517(7536):576–82. Epub 2015/01/30. doi: 10.1038/nature14129 ; PubMed Central PMCID: PMC4311405. 25631445 PMC4311405

[7] Lo Muzio L, Pannone G, Santarelli A, Bambini F, Mascitti M, Rubini C, et al. Is expression of p120ctn in oral squamous cell carcinomas a prognostic factor? Oral Surg Oral Med Oral Pathol Oral Radiol. 2013;115(6):789–98. Epub 2013/05/28. doi: 10.1016/j.oooo.2013.03.006 . 23706919

[8] Stairs DB, Bayne LJ, Rhoades B, Vega ME, Waldron TJ, Kalabis J, et al. Deletion of p120-catenin results in a tumor microenvironment with inflammation and cancer that establishes it as a tumor suppressor gene. Cancer Cell. 2011;19(4):470–83. Epub 2011/04/13. doi: 10.1016/j.ccr.2011.02.007 ; PubMed Central PMCID: PMC3077713. 21481789 PMC3077713

[9] Kidacki M, Lehman HL, Green MV, Warrick JI, Stairs DB. p120-Catenin Downregulation and *PIK3CA* Mutations Cooperate to Induce Invasion through MMP1 in HNSCC. Mol Cancer Res. 2017;15(10):1398–409. Epub 2017/06/21. doi: 10.1158/1541-7786.MCR-17-0108 . 28637905

[10] Landmesser ME, Raup-Konsavage WM, Lehman HL, Stairs DB. Loss of p120ctn causes EGFR-targeted therapy resistance and failure. PLoS One. 2020;15(10):e0241299. Epub 2020/10/29. doi: 10.1371/journal.pone.0241299 ; PubMed Central PMCID: PMC7592761. 33112928 PMC7592761

[11] Guttenplan JB, Kosinska W, Zhao ZL, Chen KM, Aliaga C, DelTondo J, et al. Mutagenesis and carcinogenesis induced by dibenzo[a,l]pyrene in the mouse oral cavity: a potential new model for oral cancer. Int J Cancer. 2012;130(12):2783–90. Epub 2011/08/05. doi: 10.1002/ijc.26344 ; PubMed Central PMCID: PMC3596885. 21815141 PMC3596885

[12] Chen KM, Guttenplan JB, Zhang SM, Aliaga C, Cooper TK, Sun YW, et al. Mechanisms of oral carcinogenesis induced by dibenzo[a,l]pyrene: an environmental pollutant and a tobacco smoke constituent. Int J Cancer. 2013;133(6):1300–9. Epub 2013/03/14. doi: 10.1002/ijc.28152 ; PubMed Central PMCID: PMC3707976. 23483552 PMC3707976

[13] Sun YW, Chen KM, Imamura Kawasawa Y, Salzberg AC, Cooper TK, Caruso C, et al. Hypomethylated Fgf3 is a potential biomarker for early detection of oral cancer in mice treated with the tobacco carcinogen dibenzo[def,p]chrysene. PLoS One. 2017;12(10):e0186873. Epub 2017/10/27. doi: 10.1371/journal.pone.0186873 ; PubMed Central PMCID: PMC5658092. 29073177 PMC5658092

[14] Chen KM, Schell TD, Richie JP Jr, Sun YW, Zhang SM, Calcagnotto A, et al. Effects of chronic alcohol consumption on DNA damage and immune regulation induced by the environmental pollutant dibenzo[a,l]pyrene in oral tissues of mice. J Environ Sci Health C Environ Carcinog Ecotoxicol Rev. 2017;35(4):213–22. Epub 2017/11/07. doi: 10.1080/10590501.2017.1391514 ; PubMed Central PMCID: PMC6130811. 29106334 PMC6130811

[15] Chen KM, Guttenplan JB, Sun YW, Cooper T, Shalaby NA, Kosinska W, et al. Effects of Black Raspberry on Dibenzo[a,l]Pyrene Diol Epoxide Induced DNA Adducts, Mutagenesis and Tumorigenesis in the Mouse Oral Cavity. Cancer Prev Res (Phila). 2017. Epub 2017/11/22. doi: 10.1158/1940-6207.CAPR-17-0278 . 29158340 PMC5839973

[16] Chen KM, Sun YW, Kawasawa YI, Salzberg AC, Zhu J, Gowda K, et al. Black Raspberry Inhibits Oral Tumors in Mice Treated with the Tobacco Smoke Constituent Dibenzo(def,p)chrysene Via Genetic and Epigenetic Alterations. Cancer Prev Res (Phila). 2020;13(4):357–66. Epub 2020/01/24. doi: 10.1158/1940-6207.CAPR-19-0496 ; PubMed Central PMCID: PMC7127947. 31969344 PMC7127947

[17] Zhang L, Gallup M, Zlock L, Basbaum C, Finkbeiner WE, McNamara NA. Cigarette smoke disrupts the integrity of airway adherens junctions through the aberrant interaction of p120-catenin with the cytoplasmic tail of MUC1. J Pathol. 2013;229(1):74–86. Epub 2012/07/27. doi: 10.1002/path.4070 ; PubMed Central PMCID: PMC4096852. 22833523 PMC4096852

[18] Guttenplan JB, Chen KM, Sun YW, Lajara B, Shalaby NAE, Kosinska W, et al. Effects of Black Raspberry Extract and Berry Compounds on Repair of DNA Damage and Mutagenesis Induced by Chemical and Physical Agents in Human Oral Leukoplakia and Rat Oral Fibroblasts. Chem Res Toxicol. 2017;30(12):2159–64. Epub 2017/10/27. doi: 10.1021/acs.chemrestox.7b00242 ; PubMed Central PMCID: PMC7880227. 29068672 PMC7880227

[19] Peiffer DS, Zimmerman NP, Wang LS, Ransom BW, Carmella SG, Kuo CT, et al. Chemoprevention of esophageal cancer with black raspberries, their component anthocyanins, and a major anthocyanin metabolite, protocatechuic acid. Cancer Prev Res (Phila). 2014;7(6):574–84. Epub 2014/03/29. doi: 10.1158/1940-6207.CAPR-14-0003 ; PubMed Central PMCID: PMC6108893. 24667581 PMC6108893

[20] El-Bayoumy K, Chen KM, Zhang SM, Sun YW, Amin S, Stoner G, et al. Carcinogenesis of the Oral Cavity: Environmental Causes and Potential Prevention by Black Raspberry. Chem Res Toxicol. 2017;30(1):126–44. Epub 2017/01/18. doi: 10.1021/acs.chemrestox.6b00306 . 28092946

[21] Lehman HL, Yang X, Welsh PA, Stairs DB. p120-catenin down-regulation and epidermal growth factor receptor overexpression results in a transformed epithelium that mimics esophageal squamous cell carcinoma. Am J Pathol. 2015;185(1):240–51. Epub 2014/12/23. doi: 10.1016/j.ajpath.2014.09.008 ; PubMed Central PMCID: PMC4278242. 25529795 PMC4278242

[22] Ferris RL. Immunology and Immunotherapy of Head and Neck Cancer. J Clin Oncol. 2015;33(29):3293–304. Epub 2015/09/10. doi: 10.1200/JCO.2015.61.1509 ; PubMed Central PMCID: PMC4586169. 26351330 PMC4586169

[23] Lee TY, Tseng YH. The Potential of Phytochemicals in Oral Cancer Prevention and Therapy: A Review of the Evidence. Biomolecules. 2020;10(8). Epub 2020/08/13. doi: 10.3390/biom10081150 ; PubMed Central PMCID: PMC7465709. 32781654 PMC7465709

[24] Wang Z, Wu VH, Allevato MM, Gilardi M, He Y, Luis Callejas-Valera J, et al. Syngeneic animal models of tobacco-associated oral cancer reveal the activity of in situ anti-CTLA-4. Nat Commun. 2019;10(1):5546. Epub 2019/12/06. doi: 10.1038/s41467-019-13471-0 ; PubMed Central PMCID: PMC6895221. 31804466 PMC6895221

[25] Guttenplan JB, Chen KM, Sun YW, Kosinska W, Zhou Y, Kim SA, et al. Effects of Black Raspberry Extract and Protocatechuic Acid on Carcinogen-DNA Adducts and Mutagenesis, and Oxidative Stress in Rat and Human Oral Cells. Cancer Prev Res (Phila). 2016;9(8):704–12. Epub 2016/06/09. doi: 10.1158/1940-6207.CAPR-16-0003 ; PubMed Central PMCID: PMC5283297. 27267891 PMC5283297

[26] Mallery SR, Tong M, Shumway BS, Curran AE, Larsen PE, Ness GM, et al. Topical application of a mucoadhesive freeze-dried black raspberry gel induces clinical and histologic regression and reduces loss of heterozygosity events in premalignant oral intraepithelial lesions: results from a multicentered, placebo-controlled clinical trial. Clin Cancer Res. 2014;20(7):1910–24. Epub 2014/02/04. doi: 10.1158/1078-0432.CCR-13-3159 ; PubMed Central PMCID: PMC3975696. 24486592 PMC3975696

[27] Mallery SR, Stoner GD, Larsen PE, Fields HW, Rodrigo KA, Schwartz SJ, et al. Formulation and in-vitro and in-vivo evaluation of a mucoadhesive gel containing freeze dried black raspberries: implications for oral cancer chemoprevention. Pharm Res. 2007;24(4):728–37. doi: 10.1007/s11095-006-9192-1 . 17372698 PMC2391087

[28] Knobloch TJ, Uhrig LK, Pearl DK, Casto BC, Warner BM, Clinton SK, et al. Suppression of Proinflammatory and Prosurvival Biomarkers in Oral Cancer Patients Consuming a Black Raspberry Phytochemical-Rich Troche. Cancer Prev Res (Phila). 2016;9(2):159–71. Epub 2015/12/25. doi: 10.1158/1940-6207.CAPR-15-0187 ; PubMed Central PMCID: PMC4764140. 26701664 PMC4764140

