# Supplementary figures and images for: Black raspberry restores the expression of the tumor suppressor p120ctn in the oral cavity of mice treated with the carcinogen dibenzo[a,l]pyrene diol epoxide
Source: PLoS One. 2021 Nov 16;16(11):e0259998. doi: 10.1371/journal.pone.0259998 (PMC8594836; doi:10.1371/journal.pone.0259998)

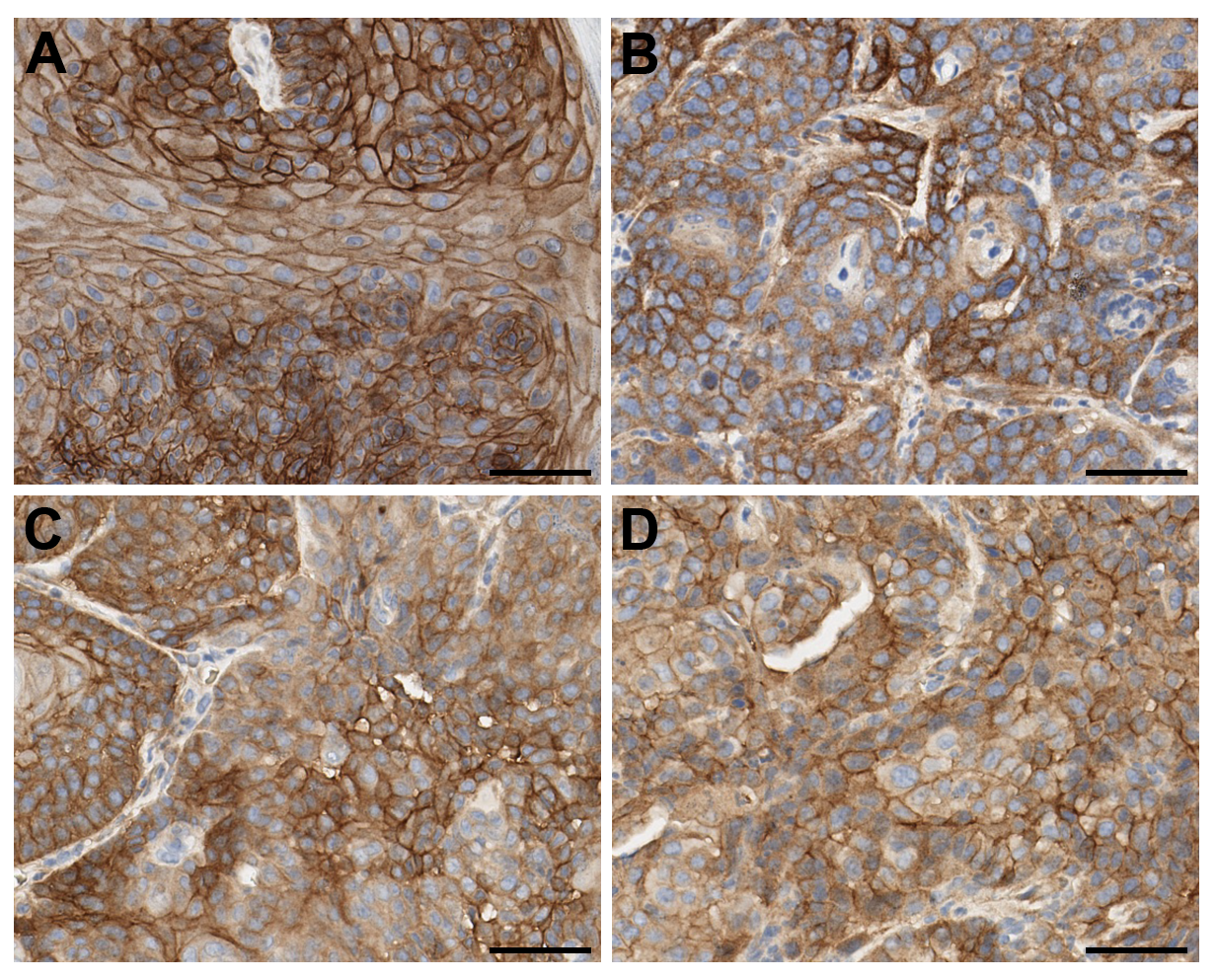

Supplement: S1 Fig — IHC staining for EGFR was performed on oral mucosal tissue samples harvested from C57Bl/6 mice treated with DBPDE with BRB (n = 10) and without BRB (n = 7) administration. Panels (A) and (C) represent papillomas while panels (B) and (D) represent invasive cancer. Mice treated with DBPDE alone, panels (A) and (B), or with BRB, panels (C) and (D), have a high EGFR expression. Of note, is the heterogeneous appearance of expression in both papillomas and invasive cancers, regardless of BRB treatment or not. Scale bar = 50uM. (TIF) [file pone.0259998.s001.tif]
